# Role of Salivary Biomarkers in Diagnosis and Detection of Dental Caries: A Systematic Review

**DOI:** 10.3390/diagnostics12123080

**Published:** 2022-12-07

**Authors:** Ahmed Alamoudi, Raghad Alamoudi, Yara Gazzaz, Aseel M. Alqahtani

**Affiliations:** 1 Department of Oral Biology, Faculty of Dentistry, King Abdulaziz University, Jeddah 21589, Saudi Arabia; 2 General Dentistry, Faculty of Dentistry, King Abdulaziz University, Jeddah 21589, Saudi Arabia

**Keywords:** caries, dental, oral, biomarkers, dental caries, salivary biomarkers, proteins, systematic review

## Abstract

Saliva plays a significant role in oral health and tooth integrity. Salivary components reduce tooth surface exposure to demineralization, protect against teeth wear and aid in enamel remineralization. There is a growing attempt to use salivary markers in diagnosing or predicting caries. However, despite the current information, there has yet to be an agreement among scholars. This study seeks to contribute more evidence on the suitability of salivary biomarkers in dental caries diagnosis. Eligible studies were electronically searched on online databases PubMed, Elsevier’s Scopus, EMBASE and Web of Science, and all the studies that met the inclusion criteria were considered. The PECOS criteria guided the study selection process based on the study question. The risk of bias was assessed using the STROBE checklist. Eighteen articles were included in the analysis. All the studies presented relevant data concerning the study objectives. There was evidence of associations between salivary biomarkers and dental caries, and the correlations were either positive or negative. The studies presented significant heterogeneity; thus, a meta-analysis was not possible. Salivary biomarkers appeared to perform crucial and complementary functions toward tooth integrity and thus may be reliable in predicting or diagnosing dental caries in patients.

## 1. Introduction

Dental caries is a highly prevalent microbial chronic dental disease that affects a greater portion of the world’s population [1]. Besides being multifactorial, dental caries is a sugar drive biofilm-mediated oral disease characterized by the phasic demineralization and demineralization of the hard tissues of the tooth [2]. The saliva surrounding the soft and hard tissues of the oral cavity includes some organic and inorganic components which contain certain factors that play a significant role for caries in the host; hence, their critical role as biomarkers in the diagnosis of caries [3]. In other words, dental caries is the result of the imbalanced cariogenic microorganisms found within the oral biofilm, which triggers the fermentation of available dietary carbohydrates to produce acids [4]. Consequently, the latter causes mineral loss on the dental hard tissues, destroying tooth structure [4]. In accordance, Sandhya et al. stated that the association between the diet, host susceptibility and microorganisms greatly determines the development of dental caries in an individual [5]. Since the teeth are consistently immersed in saliva, the saliva constituents inevitably play a fundamental role in the occurrence and progression of dental caries and are believed to be an important biomarker for caries diagnosis and detection. A biomarker is a molecule characteristic that is objectively measurable and evaluable as an indicator of a given biological or pathological process [6]. The reliable biomarkers are regarded as molecular signatures, which are heavily relied upon in assessing disease risks and in prognosis, diagnosis, or monitoring of the disease. 

The proteins within the saliva play a crucial role in maintaining the integrity of the tooth, alongside preventing the incidence of dental caries through various mechanisms [1]; for instance, inhibiting the tooth surfaces exposed to demineralization, generating the acquired enamel pellicle (AEP) to continually protect the teeth from wearing out and enabling enamel remineralization by attracting calcium ions. Nevertheless, the salivary proteins also attribute the defense of the oral cavity glycoproteins such as lactoferrin, proline-rich proteins (PRPs), carbonic anhydrase, immunoglobulins and mucins, which are ideally the most occurring, representing about 20 to 30% of the salivary proteins [7]. The mucins subcategory of MG-1 (MUC5), highly associated with high-intensity dental caries cases, is usually tasked with increasing the salivary velocity, whereas the second category, MG-2 (MUC7), is mostly found in low-intensity cases of caries, triggering the agglutination of the microorganisms within the oral cavity, including the cariogenic bacteria [8,9]. Lactoferrin, on the other hand, inhibits the production of the biofilms and growth of bacteria by attaching iron. Given its impacts on dental erosion and caries formation, the carbonic anhydrase infiltrates the dental plaque and thus neutralizes the bacterial acids bicarbonate buffer system. The cathelicidins that act as salivary antimicrobial peptides have the synergistic impact of high activity against gram-positive or gram-negative bacteria and fungi organisms, which are mainly associated with major risks of dental caries [10]. 

Given the detailed roles played by the salivary proteins in dental caries pathophysiology, there has been an increased interest in recent decades in using saliva as a diagnostic tool for dental caries. Despite positive findings from the literature, there have been numerous controversies due to the findings’ irreproducibility, primarily due to variance in methodological and statistical approaches, as well as results interpretations. For instance, the study by Piekoszewska-Ziętek et al. [11] established an association between dental caries occurrence and levels of salivary proteins in the oral cavity, contrary to the findings of Martins et al. [12] that disproved any kind of association. The latter conflicting opinions necessitate evaluating available literature evidence to enhance the understanding of this crucial developing discipline. Accordingly, the present systematic review assesses the association between salivary biomarkers or proteins and dental caries occurrence to validate the fundamental role played by the biomarkers in the detection and diagnosis of dental caries. 

## 2. Materials and Methods

### 2.1. Experimental Approach to the Review Question 

The present study was performed per the guidance of the Preferred Reporting Items for Systematic Reviews and Meta-analysis (PRISMA) statement [13]. 

### 2.2. Focused Question

Is there an established relationship between dental caries occurrence and salivary protein concentrations that substantiates the deployment of salivary biomarkers in detecting and diagnosing dental caries?

### 2.3. Eligibility Criteria 

Studies were included in this study if they met the following inclusion criteria: articles written in English between 2010 and 2022 according to PECOS (Population, Exposure, Comparator, Outcomes, and Study) design.

- Population (P): healthy individuals not under any medication that directly influences saliva composition;
- Exposure (E): salivary components as biomarkers in the dental caries incidence;
- Comparator (C): participants with dental caries (caries-active/high caries) or without caries (caries-free/low caries);
- Outcome (O): concentrations or levels of biomarkers in the study population with regard to increased (high) in the exposed group (caries-free, no caries, fewer caries) versus comparator (caries-active, dental caries patients);
- Study design (S): in vitro studies, cohort studies, cross-sectional, case-control, pilot study and longitudinal studies.

However, studies were excluded based on the following:

- Articles that did not use a predefined index for assessing dental caries in the patient groups;
- Non-English articles published earlier than 2010;
- All irrelevant publications: case reports, reviews, dissertations, unpublished articles, book chapters, reports, etc.

### 2.4. Literature Search Strategy

A comprehensive literature search was electronically performed in online databases, including PubMed, Elsevier’s Scopus, EMBASE and Web of Science, to identify articles for potential inclusion in the study. The search involved English language records published from January 2010 to September 2022, allowing the inclusion of recently published records yet to be used in other reviews. The search strategy involved the use of search terms and MeSH terms, including “dental caries”, “caries”, “saliva”, “proteins”, “biomarkers”, “stimulated saliva”, “unstimulated saliva”, “whole saliva” and other primary salivary biomarkers discussed in various studies. A search of grey literature was also performed in Google Scholar. Finally, the authors examined the reference lists of the retrieved full-text studies for potential additional articles. 

### 2.5. Study Selection

Two reviewers independently performed the article selection process. All the potential records that met the inclusion criteria were subjected to data screening to select the most relevant studies. After de-duplicating the retrieved records, the titles with keywords were manually screened, followed by abstract screening. The sources deemed relevant were considered for possible inclusion in the study synthesis. Each article was then subjected to full-text screening based on the eligibility criteria, and those that met the criteria were selected. Any discrepancies and differences between the two reviewers were settled through consensus or discussion with the third and fourth authors. 

### 2.6. Data Collection 

Data extraction was done by the two reviewers independently and then cross-checked by the third and fourth reviewers for verifying and ascertaining data accuracy. The required information was abstracted into a standardized data sheet. Any disagreements among the reviewers were solved by discussion until a consensus was reached. 

The following information was collected from the selected articles: first author’s surname and year of publication, the country where the study was conducted, study design, sample characteristics (i.e., participants’ size and age ranges), type of saliva collected for sampling, time of sample collection, methods of assessing biomarkers in the collected samples, caries index scores in the participating groups, data analysis methods, salivary parameters (biomarkers assessed), concentration levels of biomarkers identified, and main findings and inferences or conclusions. 

### 2.7. Risk of Bias Assessment

The STROBE (STrengthening the Reporting of OBservational studies in Epidemiology) [14] checklist was used to assess the selected articles’ bias risk. The checklist was based on twelve key criteria. Studies that scored eight and above were classified as low risk of bias, scores between four and seven were categorized as moderate bias risk, and those with three and below scores were considered to be a high risk of bias.

## 3. Results

### 3.1. Study Search and Selection 

**Figure 1:**
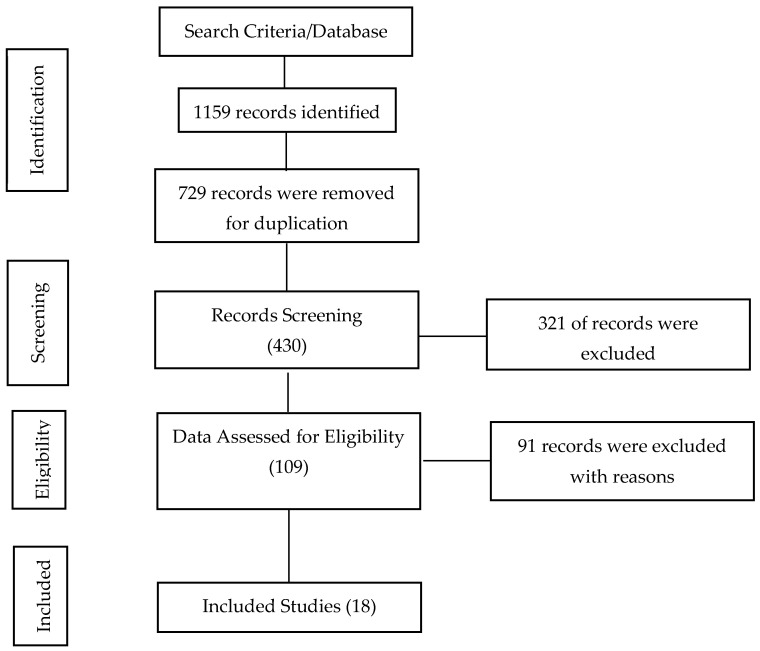
PRISMA flow diagram of search study selection process.

A total of 1159 articles were retrieved as potentially relevant records from all the databases searched. Of the total sources, 729 sources were eliminated in the deduplication process. The 430 obtained sources were screened via their titles and abstracts, and 321 articles were excluded due to non-conformity with the study objective. The remaining 109 records were subjected to full-text screening based on the predefined eligibility criteria. Ninety-one studies that did not meet the eligibility standards were excluded from the study. Finally, 18 records were certified to meet the inclusion criteria fully and thus considered for inclusion in this study. Details of the search and selection results are presented in Figure 1.

### 3.2. Primary Characteristics of Included Studies 

A detailed summary of key characteristics and findings of the 18 included studies is presented in Table 1. Four included studies were conducted in India [15,16,17,18], three studies in Poland [19,20,21], two in Brazil [22,23] and one multinational [24]. Each of the remaining seven studies were performed in China [25], Kosovo [26], Chile [27], Romania [28], Colombia [29], Egypt [30], Malaysia [31] and Iraq [32]. All the included studies were conducted within the last 12 years, but the majority were conducted between 2015 and 2021. The articles also presented a significant collective sample size. A total of 1454 participants, of both adults and children of both genders, were recruited in the 18 included studies, with the least sample size reported for a single study being 24 participants [29] and the largest being 188 participants [31]. Nevertheless, of the full sample size, 936 were minors aged between 2 years and 18 years which was reported in 11 of the included sources. Only five records presented data from the 518 adult participants aged between 18 to 50 years old. 

Contradictory results were also reported for the salivary total protein evaluated by three studies, among which two articles found a positive correlation of high protein concentration in the caries-active population [17,24] and one study [27] found a negative correlation between high levels in caries-free participants. Two studies also evaluated carbonic anhydrase, but one study found a positive correlation of high CA VI levels in the caries-active group [23], contrary to the other that presented high CA VI concentration levels for the caries-free participants over the caries-active group [30]. Similar to alpha-amylase, two studies [22,28] reported a positive association of higher enzyme concentration in caries-active group. In comparison, one noted a relatively higher concentration in caries-free participants [23], posing that alpha amylase had a protective role. The active caries group were likely to develop more caries with diminishing alpha-amylase levels. In two included studies, contradictory results were also observed for the zinc marker [17,26]. However, for proteinase 3 (PR3) [25], statherin [29,31] and LL-37 [15], a significant negative correlation between the molecules and dental caries was evident since high concentration levels were witnessed in caries-free or low caries risk participants, implying that they significantly reduced caries incidences in the caries-free groups. 

With regard to the saliva types sampled from the study population, nine included records evaluated unstimulated saliva [16,18,20,21,25,27,28,29], two studies evaluated the stimulated saliva [31,32], three studies evaluated whole saliva [15,22,23] and the remaining four studies assessed both whole unstimulated and stimulated saliva [17,24,26,30]. The saliva samples were collected mainly in the morning and afternoon hours (between 8.00 a.m. and 1.00 p.m.) for 12 studies; however, six included studies failed to state the time ranges of saliva sample collection from the participants [18,21,22,28,30,32]. The most common dental caries index was the DMF (DMFS/DMFT) used in 15 studies. Of the remaining three, one study used visual detection [28], another ICADS [29] and the last was a combination of ICADS and DMFS [23]. For the salivary biomarkers assessment, the ELISA test was used in six studies [22,23,24,25,27,29], spectrophotometry in four studies [17,26,27,28], radial immunodiffusion using the Diffu-Plate kit in two studies [18,32], the colorimetric test in two studies [16,30], the high sensitivity assays test (USCNK) [19], AGAPPE [15] and USCNK plus ELISA [20], as well as ELISA plus colorimetric kits [31]. 

### 3.3. Correlation between Salivary Biomarkers and Dental Caries 

These included studies assessed a variety of markers of salivary proteins. Among the markers, mucin, histatin, proline-rich proteins (PRP), lactoperoxidase, C-reactive proteins, cathelicidin (LL-37), immunoglobulin (IgA), albumin, statherin, salivary total protein, SOD, copper, zinc, proteinase 3 (PR3), alpha-amylase and carbonic anhydrase (CA IV) were all assessed. Although there were established correlations between dental caries pathogenicity and concentration levels of each identified biomarker, results in some markers were contradictory from the studies. The IgA was the most prevalent biomarker of all other biomolecules. Of the six studies that evaluated IgA [15,18,20,27,29,32], all except one study found a negative correlation [20], i.e., high concentration was found in the caries-free group compared with the caries-active participants, which attributed to their immense protective role in dental and oral health. Two studies [15,19] evaluated mucin, which established a positive association of high mucin levels in patients with caries prevalence or participants categorized as high caries risk or active caries group. Similar results were observed for histatin 5 [20,21], lactoperoxidase [15,20], C-reactive protein [15], SOD [17] and copper [17]. 

### 3.4. Risk of Bias Assessment of Included Studies

All the included studies reported a low risk of bias as shown in Table 2. 

## 4. Discussion

### 4.1. Summary of Main Findings 

The present study aimed to establish the fundamental role of salivary biomarkers with regard to the detection and diagnosis of the prevalence and progression of dental caries across all ages. It was achieved by evaluating for possible evidence of an association between various proteins in the oral cavity or saliva and the prevalence of caries. The articles selected for inclusion satisfied the standard STROBE quality assessment criteria. All the studies showed a correlation between the salivary biochemical markers and dental caries pathogenesis and severity levels. Generally, the study presented various biochemical markers found in saliva samples, which included the antimicrobial peptides (AMPs) molecules (cathelicidin peptide LL-37, beta-defensins, statherin and histatins), major salivary glycoproteins (proline-rich proteins (PRPs) mucins, and immunoglobulins) and minor salivary glycoproteins, which are believed to have a functionally specific contribution to the defense of the oral cavity. However, there were conflicting findings regarding the association of different molecules in this study. For instance, regardless of the statistically significant association between salivary biomarkers of the participants with or without dental caries, a section of studies reported that the concentration of particular biomarkers increased with high caries experience. Insightfully, it is worth noting that other factors, such as the size of samples, age and saliva type, impacted the comparison of results between the included studies, since the other above data varied enormously across the articles. Notwithstanding the above, the association between the salivary biochemical parameters and dental caries is crucial proof that salivary biomarkers are reliable in predicting and diagnosing dental caries.

### 4.2. Literature Insight into the Correlation Role of Biomarkers on Dental Caries Experience 

Dental caries is ideally a multifactorial condition of the teeth; thus, it cannot be rooted in a single biochemical marker to predict or diagnose disease progression or severity. According to scholars, saliva acts as the main natural defense system for the oral cavity, protecting the exposed surfaces of the tooth [33,34]. For instance, saliva can reverse tooth demineralization by its mechanical rinsing capacity or through its buffering capacity, antimicrobial activity, secretion of antimicrobial peptides or the calcium phosphate binding proteins. It can also initiate tooth remineralization with the aid of calcium, fluoride or phosphate to prevent dental caries.

Increased salivary enzymatic biomarkers, including alpha-amylase, proteinase-3 and carbonic anhydrase (CA VI), correlated with increased caries occurrence. For instance, Malawi et al. [30] and Picco et al. [22] noted increased CA VI levels in the caries-free subjects compared with the caries-active subjects. The latter is evidence toward the protective role of CA VI; for example, through the stimulation of oral buffers, pH regulation and accelerated acid neutralization. On the other hand, salivary alpha-amylase was higher in caries-active subjects than in caries-free subjects [28], as opposed to the findings of Borghi et al. [23] who noted higher amylase concentration in the caries-free subjects than in the caries-active subjects, suggesting a negative association with dental caries incidence or progression. 

Nonetheless, the literature proposes that amylase binds with the oral bacteria in the dental plaque since it helps in dietary starch hydrolysis that consequently leads to acid formation in dental plaque, accelerating the demineralization of the tooth and even dental caries progression. However, this inference is not ultimate given the conflicting opinions from the literature on amylase concentration and susceptibility of caries. 

Other than enzymatic biomarkers, antimicrobial peptides (AMPs) such as cathelicidin peptide LL-37, beta-defensins, statherin and histains also presented significant relationships with dental caries. The AMPs category of biomarkers encompasses peptides with the ability to terminate or inhibit the growth of bacteria in the oral cavity. This study reported upregulation of LL-37 in caries-free individuals [29], as well as increased histatin (HTN-5) in caries-active subjects [20]. With regard to the above extrapolations, salivary levels of AMPs can be useful in dental caries risk assessments.

The evidence presented by the included studies on correlations between dental caries and salivary glycoproteins such as mucins, immunoglobulin and proline-rich proteins is worth noting. With regard to the findings, Gabryel-Porowska et al. [19] found a correlation between increased salivary mucin (MUC1) levels and high caries experience (DMFT above 11), unlike in caries-free subjects. According to proponents, MUC1 is produced from the minor and major salivary glands, including the oral epithelial cells. Hence, it is argued to be a critical element of mucosal protection from the infection since its high concentration leads to bacterial infection of the teeth [35]. As if that was not enough, increased mucins (MUC1) levels in the oral epithelial cells are argued to be triggered by the increased inflammatory cytokines, including the interleukin-1B and interleukin-6, or the *Porphyromonas gingivalis* infection. Therefore, altered salivary cytokines levels directly influence MUC1 expression in the oral cavity. 

Regarding statherin, a study found higher levels in caries-free individuals than in the caries-active group [29]. Similar observations were made in previous studies [36,37], suggesting that statherin had a significant protective role against caries progression. In accordance, statherin depicts a strong hydroxyapatite affinity, binding calcium and maintaining the saliva’s saturation by barring spontaneous phosphate and calcium precipitation. This is what attributes protection to the tooth integrity since it enhances enamel remineralization [38]. Consistent with the above findings on antimicrobial peptides, Mandel et al. [38] agreed in their study, which noted a strong association between PRPs, statherin and histain with the absence of caries. This is attributed to the significant protective role of the phosphopeptides on the tooth structure. 

The proline-rich proteins offer an effective mechanism against dental caries progression by attaching to the *Streptococcus mutants* through the adhesion antigen and exhibiting an immunological reaction that protects the oral cavity from caries. In other words, PRPs minimize dental caries incidence by neutralizing the acidity caused by *Streptococcus mutans* bacterial functions. Considering the findings, this review also found higher concentration levels of proline-rich proteins in the caries-active group than in the caries-free individuals. The latter was contrary to previous findings that increased salivary PRPs levels were higher in caries-free than in the active study group. However, the PRP, particularly the acidic proline-rich protein, is chemically structured to trigger aminoethylpiperazine (AEP) formation, which is the main host for the bacteria responsible for dental plaque formation. Nevertheless, the acidic PRP inhibits calcium phosphate precipitation and thus boosts calcium homeostasis in the oral cavity, consequently leading to tooth enamel protection. 

On a similar account, immunoglobulins constitute humoral immunity and are also responsible for neutralizing the bacteria within the immune system mechanism, a crucial process attached to dental caries. This review established that IgA levels increased for caries-free subjects in the reviewed studies, and the findings agreed with the study by Kuriakose et al. [39]. Another study by Ranadheer et al. [40], performed on children, reported increased IgA concentration levels for the caries-free children compared with the caries-active children, suggesting a significant defensive role of IgA against *Streptococcus mutants* in a whole saliva sample. However, contrary findings were also presented in this review, noting significantly higher IgA concentration levels in caries-active subjects, similar to other studies [41,42,43]. This study suggested that the caries-free subjects experienced increased IgA levels, implying that a reduction of the IgA concentration increased the susceptibility of dental caries; hence, the reason for low concentration in the caries-active subjects. Despite the equivocal and parallel literature findings, immunoglobulins play a role in controlling dental caries development and progression. 

Moreover, this study also assessed salivary total proteins and deduced that the salivary total proteins concentrations showed a statistically critical association with dental caries experience. Again, there were contrary opinions, as reported in the study results. Nevertheless, salivary protein is crucial for protecting the tooth against desiccation. The C-reactive protein, on the other hand, was highly concentrated in caries-active subjects. Like other biomolecules, the C-reactive protein controls the immune response during inflammation development in the oral biofilms and during caries progression [44]. 

The findings on salivary albumin proteins concluded that salivary albumin controls the severity of dental plaque inflammation and underlying caries disease. It inhibits the progression of dental caries by penetrating the enamel pores to protect it against demineralization. Hence, this study found a significant negative association between salivary albumin and dental caries and a subsequent increase in caries incidence with decreased levels of salivary albumin. The latter findings were echoed by Mungia et al. [45] who reported that albumin is significantly associated with dental caries and other salivary proteins such as mucin and lysozyme. The above was also in agreement with the results of Yoshihara et al. [46] who did a cross-sectional study and reported that serum albumin levels were associated with events of dental caries. 

This study also noted increased SOD activity: higher for caries-active than for caries-free participants. Attributing to its antioxidant role, superoxide dismutase (SOD) is regarded as a detoxifying antioxidant enzyme that functions against free radicals and thus catalyzes the dis-mutation of superoxide ions to hydrogen and oxygen peroxide. 

### 4.3. Significance of the Study 

There are multiple methods that are used traditionally to detect dental caries such as a visual-tactile examination, radiographs, caries detection dyes and fiber optic transillumination (FOTI) [47]. Recently, technology offered alternative diagnostic tools, including fluorescence, electrical conductance and lasers that provide more accurate information [48]. Dentists routinely combine two diagnostic methods to detect and diagnose dental caries in order to manage it properly. However, there are disagreements on what the most accurate diagnostic method is for detecting dental caries, especially the incipient lesions [49]. Nowadays, interest toward non-invasive and personalized dentistry has been increased. Molecular assays using salivary biomarkers can be an effective tool in detecting the caries in earlier stages and assessing a patient’s caries risk [50]. This will allow a conservative and personalized approach in caries diagnosis and management for each patient. The current study identified different molecular signatures that can be used in caries diagnosis, prognosis, risk assessment and monitoring of the disease. 

### 4.4. Strengths and Limitations 

Considering a sample size of 1454 individuals, it’s arguable that this study involved a significantly large sample size which was a core strength. Besides its adherence to the PRISMA statement, this study used predetermined eligibility criteria according to PECOS, which ensured that only highly reliable studies were selected for inclusion. This was a key strength in ensuring that information was synthesized from high-quality studies to boost the reliability of the overall study. However, the study also presented some limitations worth recognizing. The included studies were mostly cohorts and cross-sectional, with one longitudinal and pilot study. In this sense, participant selection bias was inevitable, which would cause a doubly negative effect on the quality of the results. Future research would be more comprehensive by including high-quality studies with sufficient follow-up to establish conclusive information on salivary biomarkers such as dental caries detection or prognosis components. 

Nonetheless, the sample size was also a limitation. Some of the studies recruited significantly low sample sizes, which could affect the statistical power when comparing outcomes between the active and caries-free groups. Again, such information might not be of reliable standards to act as evidence for justifying salivary biomarkers as effective diagnosis components for dental caries. Finally, there was high heterogeneity among the study participants with regard to age and geographical exposure. This was an important factor to consider. Another heterogeneity factor was based on the salivary biomarkers evaluated, since some were whole evaluated saliva, unstimulated saliva stimulated or both. This contributed to a wide variation in the data obtained, which could also extend to the overall inferences of the study. 

## 5. Conclusions

This systematic review included 18 studies to produce the study results. Despite conflicting findings on the association between salivary makers and dental caries, each included study provided evidence of a relationship between components of saliva and caries. Given such a consensus, this study concludes that the salivary biomarker component concentration levels could be significant in detecting and diagnosing dental caries for both children and adults. 

## Figures and Tables

**Table 1:** Characteristics and Summary of key findings of Included Studies.

First Author, Year of Publication	Study Information and Findings
Nireeksh (2017) [15]	- Country: India. - Study design: Experimental cohort study. - Sample size: 80 patients (caries-free = 20; caries-active = 60). - Participant age: 25–40 years. - Saliva type: whole saliva. - Caries index scores: DMFT. - Sample collection time: between 10:00 a.m. and 11:00 a.m. - Data analysis: ANOVA test followed by Tukey’s post-hoc test. - Method of assessing salivary biomarkers/proteins: AGAPPE. - Saliva parameters/protein assessed: mucins, salivary proline, C-reactive protein, proteomics, salivary immunoglobulin (IgA). - Level of biomarkers: salivary total protein levels = 2.04 g/dL in caries-free vs. caries-active (group I (1.71 g/dL), group II (0.82 g/dL) and group III (0.51 g/dL). - Main finding: Study reported high levels of salivary total protein, salivary IgA and salivary albumin globulin in caries-free group, while mucin and salivary C-reactive protein were high in caries-active group. - 95% proline were in caries-free group, whereas caries-active group showed 65% proline protein bands. - Conclusion: There were both positive and negative correlations between the salivary biomarkers and outcomes on dental caries; thus, they can play important role in detecting the onset and progression of dental caries.
Khandelwa (2019) [16]	- Country: India. - Study design: Experimental cohort study. - Sample size: 60 healthy adults: Group 1: caries-free (DMFT 0) = 15; experimental: Group 2 (DMFT 1–5) = 15; Group 3 (DMFT 6–10) = 15; Group 4 (DMFT above 10) = 15. - Participant age: 18–40 years. - Saliva type: unstimulated saliva. - Caries index scores: DMFT. - Sample collection time: between 10:00 a.m. and 11.00 a.m. - Data analysis: One-way ANOVA and Tukey’s honestly significant difference test. - Method of assessing salivary biomarkers/proteins: Bromocresol green method (albumin colorimetric test). - Saliva parameters/protein assessed: salivary albumin. - Level of biomarkers: salivary albumin levels for the groups 1, 2, 3 and 4 were 0.086 ± 0.009 mg/mL, 0.083 ± 0.006 mg/mL, 0.070 ± 0.008 mg/mL and 0.056 ± 0.009 mg/mL, respectively. - Main finding: There was an increase in caries incidence with decreased albumin levels. - Conclusion: The correlation suggests that salivary albumin is crucial for maintaining tooth integrity; hence, it is implied that the biomarker could be used as a detection and prognostic tool for tooth decay.
Murugeshappa (2018) [24]	- Country: India, Malaysia and China. - Study design: Experimental cohort study. - Sample size: 70 subjects (caries-free = 35; caries-active = 35). - Participant age: 7–12 years. - Saliva type: Stimulated and unstimulated saliva. - Caries index scores: DMFT. - Sample collection time: between 10 a.m. and 1 p.m. - Data analysis: Nonparametric testing of hypothesis using the Mann–Whitney test and independent *t*-test. - Method of assessing salivary biomarkers/proteins: ELISA & Cayman protein determination kits. - Saliva parameters/protein assessed: salivary immunoglobulins and salivary total proteins. - Level of biomarkers: salivary IgA = 0.079086 mcg/mL (caries-active group) vs. 0.114286 mcg/mL (caries-free group); salivary total proteins = 2.71 (caries-active) vs. 1.8 (caries-free). - Main finding: Study reported a positive association of caries and salivary total protein and a negative correlation with salivary IgA. - Conclusion: The correlation between caries and biomarkers implied that the biomarkers can play important roles in detecting and diagnosing dental caries.
Hegde (2014) [17]	- Country: India. - Study design: Experimental cohort study. - Sample size: 80 healthy patients: 20 caries-free (DMFT = 0); 60 caries-active (DMFT above 10). - Participant age: 25–50 years. - Saliva type: Stimulated and unstimulated saliva. - Caries index scores: DMFT. - Sample collection time: Noon. - Data analysis: Student *t*-test. - Method of assessing salivary biomarkers/proteins: Atomic nitro blue tetrazolium chloride reduction method and absorption spectrophotometry. - Saliva parameters/protein assessed: superoxide dismutase (SOD) activity/total protein, copper and zinc levels. - Level of biomarkers: total protein; caries-free group vs. caries-active = 0.19 ± 0.049, vs. 0.35 ± 0.139; copper = 0.237 ± 0.051 vs. 0.511035 ± 0.096; zinc = 0.93 ± 0.50 vs. 1.169 ± 0.21. - Main finding: Study showed increase in copper and zinc levels in the caries-active group vs. the caries-free group, as well as increased SOD activity. - Conclusion: The concentration of total salivary protein, copper and zinc increased with dental caries/decays, implying that the cofactors could be used as noninvasive indicator correlating diagnosis and progression of caries.
Gabryel-Porowska (2014) [19]	- Country: Poland. - Study design: Experimental cohort study. - Sample size: 35 patients (control (DMF = 3) = 8; research group (DMF >11) = 27 - Participant age: 18 years. - Saliva type: Unstimulated whole saliva. - Caries index scores: DMFT. - Sample collection time: between 9:00 and 11:00 a.m. - Data analysis: Mann-Whitney nonparametric U test and Shapiro-Wilk test/*t*-test. - Method of assessing salivary biomarkers/proteins: high sensitivity assay kits (USCNK). - Saliva parameters/protein assessed: Mucins (MUC5B, MUC7, and MUC1). - Level of biomarkers: MUC5B (ng/mL); 0.05 (0.05–0.78) control vs. rsch. 0.70 (0.06–2.34). MUC7 (ng/mL); 0.17 (0.06–0.32) control vs. rsch 0.13 (0.06–0.42). MUC1 = 0.17 ± 0.05 contrl vs. rsch. = 0.24 ± 0.08. - Main finding: MUC1 proteins and MUC5B protein levels were higher in research grp than control grp, while MUC7 showed slight statistical insignificant decrease. - Conclusion: Study suggests a correlation of salivary mucins (MUC1 & MUC5B) and dental caries prevalence, implying that the saliva biomarkers were effective for detection and diagnosis of caries.
Yang (2015) [25]	- Country: China. - Study design: Experimental study. - Sample size: 128 healthy children: no dental caries (NDC) (DMFT = 0) = 46; low dental caries (LDC) (DMFT 1–4) = 49; high dental caries (HDC) (DMFT 5–15) = 33. - Participant age: 6 years. - Saliva type: Whole, unstimulated saliva. - Caries index scores: DMFT. - Sample collection time: Between 8:00 and 10:00. - Data analysis: one-way analysis of variance (ANOVA). - Method of assessing salivary biomarkers/proteins: ELISA kit/test. - Saliva parameters/protein assessed: Saliva proteinase 3 (PR3). - Level of biomarkers: PR3 for the NDC group (17.82 ± 7.31) ng·mL^−1^, LDC group (12.79 ± 6.19) ng·mL^−1^; HDC group (11.07 ± 7.10) ng·mL^−1^. - Main finding: Study reported significant decrease of PR3 with the increase of dental caries severity indicating a correlation of negative association of dental caries and salivary PR3 concentration. - Conclusion: The negative correlation suggested that PR3 can be used in detecting and diagnosing caries prevalence.
Monea (2018) [28]	- Country: Romania. - Study design: Experimental cohort study. - Sample size: 142 children. - Participant age: 9–12 years. - Saliva type: Unstimulated saliva. - Caries index scores: Visual detection of dented caries. - Sample collection time: N/R. - Data analysis: GraphPad Prism 7.03 and the Mann-Whitney test. - Method of assessing salivary biomarkers/proteins: spectrophotometer. - Saliva parameters/protein assessed: salivary alpha-amylase. - Level of biomarkers: Caries-free = 150.53 ± 2.45 U/mL or 147.28 ± 2.1 U/mL, caries-active = 156.83 ± 1.59 U/mL or 158.18 ± 2.41 U/mL. - Main finding: Caries-active had higher salivary enzyme (alpha-amylase) than caries-free. - Conclusion: Saliva can be considered as noninvasive diagnostic fluid through the correlation of salivary alpha amylase biomarker and risks of dental caries.
Picco (2017) [22]	- Country: Brazil. - Study design: Cross-sectional. - Sample size: 74 children (caries-free = 37; caries-active = 37). - Participant age: 7–9 years. - Saliva type: whole saliva. - Caries index scores: DMFT. - Sample collection time: N/R. - Data analysis: Student’s *t*-test and the Mann–Whitney test. - Method of assessing salivary biomarkers/proteins: ELISA and zymography. - Saliva parameters/protein assessed: Carbonic anhydrase (CA) VI. - Level of biomarkers: The activity of this isoenzyme or CA VI was higher in caries group (3391 ± 2046, *n* = 37) than in the caries-free (1383 ± 1076, *n* = 37). - Main finding: Salivary CA VI level was low in the saliva of caries-free children. However, salivary CA VI activity was higher in caries-active group, implying that dental caries was affected by CA VI activity. - Conclusion: Salivary CA VI was a potential biomarker for diagnosis and detection of dental caries.
Angarita-Díaz (2021) [29]	- Country: Colombia. - Study design: Pilot study. - Sample size: 24 children (caries-free = 12; caries-active = 12). - Participant age: 6 to 12 years. - Saliva type: Unstimulated saliva. - Caries index scores: ICDAS. - Sample collection time: between 8 a.m. and 11 a.m. - Data analysis: The Mann–Whitney U test and Kruskal–Wallis test. - Method of assessing salivary biomarkers/proteins: ELISA. - Saliva parameters/protein assessed: IgA, fibronectin, cathelicidin LL-37, and statherin. - Level of biomarkers: IgA concentration for caries-free vs. caries-active children = 48,250 vs. 37,776, LL-37 = 56.1 vs. 46.3, Stetherin = 93,199.1 vs. 94,734.6. - Main finding: Higher statherin levels were reported in caries-free than caries-active; slightly higher IgA and lower format concentrations were reported in caries-free group. - Conclusion: The marker correlated significantly with caries thus can be deployed in detection and diagnosis of caries progression.
Pateel (2022) [31]	- Country: Malaysia. - Study design: Cross-sectional study. - Sample size: 188 healthy adults (DMFT = 0/caries-free = 29; caries-active = 159). - Participant age: 18 and 50 years. - Saliva type: Stimulated whole saliva. - Caries index scores: DMFT. - Sample collection time: between 10:00 a.m. and 1:00 p.m. - Data analysis: SPSS (Spearman’s rho and Pearson’s correlation coefficient). - Method of assessing salivary biomarkers/proteins: ELISA Kit and calorimetric assay kit. - Saliva parameters/protein assessed: Statherin, Proline-rich proteins (PRP), Calcium. - Level of biomarkers: PRP statistical correlation = *r* = 0.025; *P* = 0.733; calcium = *r* = 0.015, *P* = 0.841 Statherin showed an inverse relation; declining trend with increase in caries levels. - Main finding: There was no statistically significant correlation between PRP, Calcium and dental caries progression. - Conclusion: The inverse correlation between statherin and PRP levels suggests their roles in caries progression, hence can be used to diagnose or predict dental caries incidences.
Gornowicz (2014) [20]	- Country: Poland. - Study design: Experimental cohort study. - Sample size: 35 adolescents (control = 8 (DMF = 3); experimental = 27 (DMF = above 11)). - Participant age: 18 years. - Saliva type: Unstimulated whole saliva. - Caries index scores: DMFT. - Sample collection time: between 9:00 a.m. and 11:00 a.m. - Data analysis: Mann–Whitney U nonparametric test. - Method of assessing salivary biomarkers/proteins: High-sensitivity assay kit (USCNK) and ELISA. - Saliva parameters/protein assessed: sIgA, histatin-5, and lactoperoxidase (LPO) - Level of biomarkers: sIgA (mg/dL) = 8.10 (2.1–22.2) in control group vs. 13 (7.2–23.8) in experimental group. Histatin-5 (ng/mL) = 16.89 (14.11–29.95) in control group vs. 66.84 (14.11–649) in experimental group. LPO (nmol/L) = 2148 (1305–3248) in control group vs. 3047 (1515–3313) in experimental group. - Main finding: The experimental group (DMF above 11) showed increasing levels of sIgA, histatin-5 and lactoperoxidase vs. the control group. It suggests the presence of caries associated with increased levels of salivary biomarkers positively. - Conclusion: sIgA, histatin-5 and lactoperoxidase markers strongly correlated with increased caries; hence, they can potentially diagnose and predict dental caries incidence and progression.
Jurczak (2015) [21]	- Country: Poland. - Study design: Experimental cohort study. - Sample size: 82 pediatric patients (ECC caries-free/control = 41; patients with ECC/experimental = 41). - Participant age: N/R. - Saliva type: Unstimulated saliva. - Caries index scores: DMFT. - Sample collection time: N/R. - Data analysis: *t*-test and one-way ANOVA. - Method of assessing salivary biomarkers/proteins: ELISA. - Saliva parameters/protein assessed: β-defensin-2 and histatin-5. - Level of biomarkers: salivary HST-5 and β-defensin-2 levels in patients with ECC (50.75 ± 2.11; 2.29 ± 0.05 ng/mL, respectively) were significantly increased compared with control (healthy subject) (15.29 ± 1.16; 2.15 ± 0.07 ng/mL). - Main finding: Significant increase of histatin-5 and β-defensin-2 concentrations were noted in experimental than control group, which correlated with caries progression. - Conclusion: The changes in concentrations of the saliva proteins during dental caries progression implies that they can be used to detect and diagnose caries onset or progression.
Borghi (2016) [23]	- Country: Brazil. - Study design: longitudinal study. - Sample size: 100 children (caries-free = 55; caries-active = 45). - Participant age: 24 and 48 months. - Saliva type: whole saliva. - Caries index scores: DMFS and DMFT. - Sample collection time: between 8:00 and 10:00 a.m. - Data analysis: Shapiro–Wilk test, Mann–Whitney, Spearman correlation. - Method of assessing salivary biomarkers/proteins: ELISA kit and zymography. - Saliva parameters/protein assessed: Alpha amylase, carbonic anhydrase VI. - Level of biomarkers: Alpha amylase = 99.2 ± 84 for caries-free group and 55.6 ± 52.8 for caries-active group. CA VI activity = 0.250 ± 43 for caries-free group and 0.31 ± 0.65 for caries-active group. - Main finding: CA VI was significantly higher in caries-active group. Salivary amylase was significantly higher in caries-free group. Hence, caries-active (low alpha amylase) were more likely to develop ECC than caries-free. - Conclusion: The negative correlation of the markers (alpha amylase) and dental caries implied that the biomarker could significantly predict or diagnose increased risk of dental caries progression.
Makawi (2017) [30]	- Country: Egypt. - Study design: Experimental cohort study. - Sample size: 120 children (caries-free/low caries risk = 60; caries-active/high caries risk = 60). - Participant age: 3–5 years and 13–15 years of age group. - Saliva type: stimulated and non-stimulated. - Caries index scores: DMFT and dmft. - Sample collection time: N/R. - Data analysis: one-way ANOVA followed Tukey’s post-hoc test. - Method of assessing salivary biomarkers/proteins: Ericsson method and Colorimetric ab65622 (abcam). - Saliva parameters/protein assessed: carbonic anhydrase (CA VI) and phosphate buffer activity. - Level of biomarkers: CA IV = 3.87 ± 1.47 in caries-active group vs. 6.75 ± 0.44 caries-free group. Phosphate buffer = 5.94 ± 1.76 for caries-free vs. 3.84 ± 0.42 in caries-active group. - Main finding: Higher levels of C.A. were detected in dental free/low caries group compared to caries-active/higher caries group. Similar results were observed for phosphate buffer concentrations/activity for caries-free group superior vs. the caries-active group. - Conclusion: The negative correlation of phosphate buffer activity and C.A. depicted that they were potential biomarkers for detecting dental caries progression.
Doifode (2011) [18]	- Country: India. - Study design: Experimental cohort study. - Sample size: 30 children (caries-free = 15; caries-active = 15). - Participant age: 8–10 years. - Saliva type: Unstimulated whole saliva. - Caries index scores: DMFS. - Sample collection time: N/R. - Data analysis: Unpaired *t*-test. - Method of assessing salivary biomarkers/proteins: radial immunodiffusion method using Diffu-Plate kit. - Saliva parameters/protein assessed: salivary IgA. - Main finding: IgA levels for caries-free group = 10.74 mg/dl ± 1.52; caries-active group = 8.98 mg/dl ± 1.56. - Level of biomarkers: salivary IgA were higher in caries-free group than caries-active group; thus, it played a crucial protection role as immunological control against caries. - Conclusion: The negative correlation between dental caries and salivary IgA signified that the IgA marker could potentially be used to predict or diagnose caries progression.
Heba (2016) [32]	- Country: Iraq. - Study design: Experimental cohort study. - Sample size: 60 healthy children (caries-free = 30; caries-active = 30). - Participant age: 7 to 10 years. - Saliva type: stimulated saliva. - Caries index scores: DMFT and dmft. - Sample collection time: N/R. - Data analysis: SPSS, ANOVA. - Method of assessing salivary biomarkers/proteins: radial immunodiffusion method using immunodiffusion plate (Diffu-Plate kit). - Saliva parameters/protein assessed: immunoglobulin IgA. - Level of biomarkers: IgA levels = 127.29 ± 28.14 or 130.52 ± 29.17 for caries-free; 112.22 ± 31.33 or 103.77 ± 24.20 for caries-active. - Main finding: Salivary IgA was higher in caries-free than caries-active, implying that IgA had a protection role against dental caries. - Conclusion: The negative correlation between dental caries and IgA levels signified a prognostic role of the biomarker in detecting dental caries prevalence and progression.
Sejdini (2018) [26]	- Country: Kosovo. - Study design: cross-sectional study. - Sample size: 106 school children (caries-free = 25; caries-active = 81). - Participant age: 12–13 years. - Saliva type: stimulated and unstimulated whole saliva. - Caries index scores: DMFT. - Sample collection time: Morning (8 a.m. to 10 a.m.). - Data analysis: Statistics for Windows/Release 7.0. - Method of assessing salivary biomarkers/proteins: Spectrometer analysis. - Saliva parameters/protein assessed: Zinc salts (Zn). - Level of biomarkers: Zn in caries-free = 6.72 mmol/L vs. caries-active group = 0.07 mmol/L. - Main finding: Zn concentration significantly increased in caries-free group vs. caries-active, showing a positive reduction of caries incidence. - Conclusion: The positive association of Zn with caries reduction implied that it can be used to detect and diagnose caries prevalence risks and progression.
Castro (2016) [27]	- Country: Chile. - Study design: Experimental cohort study. - Sample size: 40 participants (caries-free = 20; caries-active = 20). - Participant age: 22–28 years old. - Saliva type: Unstimulated saliva. - Caries index scores: ICADS and DMFT. - Sample collection time: between 9:00 and 11:00 a.m. - Data analysis: Student’s *t*-test. - Method of assessing salivary biomarkers/proteins: Bradford method with spectrophotometer and Western blotting. - Saliva parameters/protein assessed: salivary IgA & total protein concentrations. - Level of biomarkers: Total protein concentration in caries-free = 50.65 ± 7.5 μg/mL; caries-active = 26.80 ± 2.5 μg/mL. IgA levels = 11.27 ± 0.5 μg in caries-free group vs. 1.71 ± 0.2 μg in caries-active group. - Main finding: Total protein concentration was higher in caries-free than caries-active. However, caries-free reported higher IgA concentration than caries-active. - Conclusion: The correlation of caries and salivary IgA implies that it can be used to diagnose dental caries risks and progression.

Decayed, missing, filled teeth (DMFT); decayed, missing, filled surfaces (DMFS); Statistical Package for Social Sciences (SPSS); enzyme-linked immunosorbent assay (ELISA); International Caries Detection and Assessment System (ICDAS)-certified dentist (kappa value ≥ 0.7); not reported (N/R); early childhood caries (ECC).

**Table 2:** STROBE Risk of Bias Assessment of Included Studies. (x: presence of criteria).

Checklist Item	Nireeksh (2017) [15]	Khandela (2019) [16]	Muruge-shappa (2018) [24]	Hegde (2014) [17]	Gabryel-Porowska (2014) [19]	Yang (2015) [25]	Monea (2018) [28]	Picco (2017) [22]	Angarita-Díaz (2021) [29]	Pateel (2022) [31]	Gornowiz (2014) [20]	Jurczak (2015) [21]	Borghi (2016) [23]	Makawi (2017) [30]	Doifode (2011) [18]	Heba (2016) [32]	Sejdini (2018) [26]	Castro (2016) [27]
Inclusion criteria	X	X	X	X	X	X	X	X	X	X	X	X	X	X	X	X	X	X
Exclusion criteria	X	X	X	X	X	X	X	X	X	X	X	X	X	X	X	X	X	X
No fluoride exposure during tooth development																		
Dental caries diagnosis criteria	X	X	X	X	X	X	X	X	X	X	X	X	X	X	X	X	X	X
Radiographic exam																		
Experienced calibrated examiner	X	X	X	X	X	X	X	X	X	X	X	X	X	X	X	X	X	X
Salivary collection description	X	X	X	X	X	X	X	X	X	X	X	X	X	X	X	X	X	X
Salivary analysis description	X	X	X	X	X	X	X	X	X	X	X	X	X	X	X	X	X	X
Statistical analysis description	X	X	X	X	X	X	X	X	X	X	X	X	X	X	X	X	X	X
Paired groups	X	X	X	X	X	X	X	X	X	X	X	X	X	X	X	X	X	X
Blinded study																		
Risk of bias score	Low	Low	Low	Low	Low	Low	Low	Low	Low	Low	Low	Low	Low	Low	Low	Low	Low	Low

## Data Availability

Not applicable.
